# Design of Lipopeptides and dsRNA‐Lipopeptide Complexes for Sprayable RNAi‐Based Plant Protection

**DOI:** 10.1002/psc.70133

**Published:** 2026-09-25

**Authors:** Pascal Geisler, Frank Steiniger, Andreas Vilcinskas, Christoph Hellmann

**Affiliations:** ^1^ Fraunhofer Institute for Molecular Biology and Applied Ecology IME Giessen Germany; ^2^ Electron Microscopy Center, Jena University Hospital Friedrich Schiller University Jena Jena Germany; ^3^ Institute of Insect Biotechnology Justus‐Liebig‐University Gießen Germany

**Keywords:** dsRNA formulation, dsRNA‐lipopeptide complexes, lipopeptide surfactants, microfluidic mixing, RNA interference, spray‐induced gene silencing, targeted pest insect control

## Abstract

Broad‐spectrum insecticides persist in the environment, affecting non‐target organisms and selecting for resistant pest populations. In contrast, RNA interference (RNAi) achieves species‐targeted pest control triggered by the delivery of exogenous double‐stranded RNA (dsRNA), which is biodegradable and broken down into harmless components by nucleases. Sprayable applications require suitable formulation strategies for dsRNA protection during delivery. Here, we describe the preparation of dsRNA‐lipopeptide complexes (LPCs) composed of non‐toxic short‐chain lipopeptide surfactants by microfluidic mixing, yielding positively charged dispersions of LPCs < 200 nm in diameter, which encapsulate and protect dsRNA from enzymatic degradation. Some lipopeptides were found to undergo thermo‐reversible gelation, self‐assembling to fibrillar nanostructures, but remained effective for dsRNA delivery to induce mortality in Colorado potato beetle larvae feeding on sprayed plants. Our results show that lipopeptide surfactants are suitable for the formulation of dsRNA to enable effective delivery to pest insects.

## Graphical abstract

Lipopeptide surfactants self‐assemble into nanostructure carriers that complex, protect, and deliver dsRNA, establishing structure–function relationships for next‐generation RNAi‐based insect control.

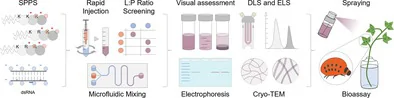

## 1 Introduction

The large‐scale cultivation of crops is necessary for global food security [1, 2, 3]. However, the damage caused by pest insects and the pathogens they transmit is estimated to cost billions of US$, threatening not only food security but also the agricultural economy [4, 5, 6]. Climate change is predicted to accelerate the growth of insect pest populations and their expansion to new geographical areas, making infestations more difficult to control [7, 8, 9]. Pest management strategies largely rely on the intensive use of broad‐spectrum chemical insecticides, but this is associated with negative impacts on the ecosystem, such as off‐target effects in beneficial insects and the loss of biodiversity [10, 11, 12, 13, 14, 15]. The accumulation of pesticide residues increases the risk of negative long‐term effects throughout the food chain [16, 17, 18, 19]. Moreover, the widespread use of chemical insecticides creates selection pressure for resistant pest populations. For the Colorado potato beetle *Leptinotarsa decemlineata* (CPB), the Arthropod Resistance Database lists more than 380 reports of field‐evolved resistance to neonicotinoids and other agents [20, 21, 22].

RNA interference (RNAi) is a post‐transcriptional gene silencing mechanism triggered by double‐stranded RNA (dsRNA) that offers a promising biotechnological alternative to conventional pesticides by restricting effects to the target species and thus minimizing the impact on beneficial insects and other non‐target organisms [23, 24, 25, 26, 27, 28]. The application of dsRNA by spraying ensures sufficient exposure but excess dsRNA in agricultural fields is broken down by enzymes and non‐enzymatic processes within days, suggesting a low risk of bioaccumulation [29, 30]. The potential of RNAi, in the form of spray‐induced gene silencing (SIGS), has recently been demonstrated by Greenlights first‐in‐class plant protection product *Calantha*, which targets the PSMB5 proteasome subunit in CPB [31, 32, 33]. However, the rapid degradation of unprotected dsRNA can limit the efficacy of SIGS, and formulation strategies are therefore required for transient protection to ensure dsRNA delivery [34]. Lipid nanoparticles offer a versatile and powerful formulation platform for delivery of dsRNA for agricultural use [35, 36, 37, 38], and simple lipopeptide surfactants that can be manufactured in solution (rather than by solid‐phase synthesis) are a suitable materials class for further formulation optimizations [39, 40, 41]. These biodegradable amphiphilic molecules are well known for their low toxicity, but they have not yet been widely used for the development of agricultural dsRNA formulations [42, 43, 44, 45, 46, 47]. Lipopeptides can easily be tailored to match physicochemical performance criteria, and to incorporate components that enhance cellular uptake or enable targeted delivery [48, 49, 50, 51, 52].

Here, we synthesized three surfactants by combining an *N*,*N*′‐dilauroyl lysine hydrophobic tail with different amino acid head groups composed of lysine and arginine, offering cationic side chain functions under neutral pH conditions. We used microfluidic mixing to prepare dsRNA–lipopeptide surfactant complex (LPC) formulations, which protect their cargo against enzymatic digestion. Finally, we demonstrated the delivery of insecticidal dsRNA to CPB feeding on plants sprayed with the LPC formulations. Our results show that lipopeptide surfactants can be used to formulate sprayable LPCs that enable the effective delivery of dsRNA to pest insects.

## 2 Materials and Methods

### 2.1 Chemicals

Resin, coupling reagents and Fmoc amino acids used for solid‐phase peptide synthesis (SPPS) were purchased from Carbolution. Other chemicals and solvents were purchased from VWR International. Ultra‐pure water was produced using a Sartorius Arium Pro purification system. We acquired dsRNA from Genolution. The sequences of dsGFP and dsNCM (used as negative and positive controls for insecticidal activity, respectively) are provided in the Supporting Information.

### 2.2 Solid‐Phase Peptide Synthesis

Before SPPS, 2‐chlorotrityl chloride resin (1 g, 1.085 mmol/g) was refluxed with thionyl chloride (1.2 eq.) and pyridine (2.4 eq.) in dichloromethane (DCM; 10 mL) for 2 h. The resin was filtered off, washed with DCM several times and dried *in vacuo* overnight. SPPS was carried out as shown in Table S1 (for LC–MS analysis, see Figures S1–S3) [53]. Briefly, the first amino acid (1.5 eq.) was coupled to the resin (0.1 g) using *N*,*N*‐diisopropylethylamine (DIPEA; 3.0 eq.) in DCM (2 mL, 90 min) and Fmoc cleavage was carried out with piperidine in 20% v/v dimethylformamide (DMF; 2 × 4 mL, 5 min). The coupling of subsequent Fmoc amino acids and the final double‐endcapping with lauric acid was achieved using 1‐[*bis* (dimethylamino)methylene]‐1*H*‐1,2,3‐triazolo[4,5‐*b*]pyridinium 3‐oxide hexafluorophosphate (HATU), DIPEA and 1‐hydroxybenzotriazole (HOBt) in the ratio 3.0:6.0:0.1 eq. in DMF (2 mL, 60 min each). Cleavage from the resin and global deprotection was achieved using a mixture of trifluoroacetic acid (TFA), water and triisopropylsilane (TIS) in the ratio 95:2.5:2.5 v/v (2 × 5 mL, 30 min each). The TFA was co‐evaporated with DCM, and the products were isolated from the lyophilized residue by semi‐preparative HPLC using an Agilent 1200 device with a Nucleodur C18 Gravity SB column (250 × 10 mm, 3 μm) and solvents A (H_2_O + 0.1% v/v formic acid) and B (acetonitrile + 0.1% formic acid) in the following program: 0 min 60% A, 5 min 60% A, 15 min 25% A, 15.1 min 0% A, 23 min 0% A, 23.1 min 60% A, and 30 min 60% A.

### 2.3 UPLC‐ESI‐qQ‐TOF High‐Resolution Tandem Mass Spectrometry

Ultra‐high performance liquid chromatography high‐resolution tandem mass spectrometry (UPLC‐HR‐MS/MS) was carried out using an Agilent Infinity II UPLC system equipped with an Acquity UPLC BEH C18 column (100 × 2.1 mm, 1.7 μm) and an Acquity UPLC BEH C18 VanGuard pre‐column (5 × 2.1 mm, 1.7 μm), both from Waters, connected to a DAD detector and a MaXis II (Bruker) mass spectrometer equipped with an electrospray ionization source. MS spectra were calibrated using 1.25‐mM sodium formate in a mixture of ultra‐pure water and isopropanol (1:1 v/v) as the internal standard. Analytical separations were carried out using a gradient of solvent A (water +0.1% formic acid) and B (acetonitrile +0.1% formic acid) with the following program: 0 min 95% A, 0.8 min 95% A, 18.7 min 4.75% A, 18.8 min 0% A, 28 min 0% A, 28.1 min 95% A, and 30 min 95% A and a column oven temperature of 45°C at a flow rate of 0.6 mL/min. Acquired spectra were processed using Bruker DataAnalysis v6.2.

### 2.4 Lipid‐to‐Phosphate Ratio of dsRNA‐LPC Formulations

The lipid‐to‐phosphate (L:P) ratio was screened via the rapid solvent syringe injection of lipopeptides (dissolved in ethanol) into aqueous dsRNA solutions [54]. For each L:P ratio, we prepared individual aliquots of aqueous dsGFP solutions (1.8 mL in 15‐mL tubes, 0.111 mg/mL) and ethanolic lipid blends consisting of the synthesized lipopeptide and the PEGylated lipid Kolliphor HS 15 (Sigma‐Aldrich) as a dispersion stabilizer (0.2 mL in 1.5‐mL tubes, stoichiometry listed in Table S2). All aliquots were equilibrated at 65°C for 30 min, after which the dissolved lipid blends were aspirated into 1‐mL syringes and quickly dispensed into the center of the vortex (created by a vortex mixer at 300 rpm) of the dsRNA solutions. Vortexing was continued for 30 s after injection and formulations were then cooled to room temperature. All formulations were equilibrated at 4°C overnight before further analysis. Dynamic light scattering (DLS) results are summarized in Table S2 (also see Figures S4 and S5).

### 2.5 Microfluidic Mixing

The dsGFP‐LPC and dsNCM‐LPC formulations were prepared using a Fluidic 1079 3D serpentine microfluidic mixer chip (Microfluidic Chip Shop) as previously described (Table S3) [38]. Briefly, aqueous dsRNA solutions (10 mL, 0.111 mg/mL) and ethanolic lipid blends (2 mL, stoichiometry listed in Table S4) were equilibrated at 65°C for 30 min and then rapidly aspirated (bubble‐free) into 10‐mL Luer‐lock syringes. The syringes were connected to the mixing setup and mounted to syringe pumps. Aqueous solutions were delivered at 10.8 mL/min to the outer inlet channels and ethanolic solutions at 1.2 mL/min to the central inlet channel of the chip, resulting in a total flow rate of 12 mL/min and a 9:1 flow rate ratio. Eluted dsRNA‐LPC formulations were collected in 2‐mL fractions until the aqueous phase was fully delivered. Collected fractions were allowed to come to room temperature and equilibrated at 4°C overnight before further analysis. DLS results are summarized in Table S4 (see also Figures S7 and S8).

### 2.6 Agarose Gel Electrophoresis

For the analysis of unformulated dsRNA and dsRNA‐LPC formulations, samples were separated by 2% (w/v) agarose gel electrophoresis in TAE buffer (40‐mM Tris–HCl, 20‐mM glacial acetic acid, 1‐mM EDTA, and pH 8.4) for 45 min at 80 V. Gels were prepared from NEEO ultra‐quality agarose (Carl Roth). Samples of unformulated dsRNA or dsRNA‐LPC formulations (7 μL) were mixed with 1‐μL ultra‐pure water, 1‐μL 10 × BlueJuice loading dye (Thermo Fisher Scientific), and 1‐μL 3 × SYBR Gold in DMSO (Thermo Fisher Scientific). Decomplexation was achieved by mixing with 1‐μL 1% SDS instead of water, and incubation at room temperature for 20 min. Results were visualized using the GelDoc XR + system and ImageLab software.

### 2.7 RNase III Assay

Protection against RNase III was tested by dilution of either unformulated dsRNA or dsRNA‐LPC formulations (20 μL, 0.1 μg/μL) with ultra‐pure water (24 μL) and mixing with ShortCut RNase III (1 μL, 2 U/μL; New England Biolabs) and 10 × manganese(II) chloride (5 μL, supplied with enzyme). The reaction mixtures were incubated at 37°C shaking at 500 rpm for 24 h and were then quenched by adding 5‐μL proteinase K (0.8 U/μL; New England Biolabs) and incubation for 1 h. Results were assessed by agarose gel electrophoresis as described above.

### 2.8 Encapsulation Efficiency

Encapsulation efficiency was determined according to established procedures [55]. Samples containing dsRNA‐LPC formulations (1 μL, 0.1 μg/μL) were mixed by pipetting with 99‐μL ultra‐pure water or 1% Triton X‐100 and 100‐μL 50 × SYBR Gold. The mixtures were transferred to a black, flat‐bottomed 96‐well plate (Greiner), incubated for 5 min at room temperature and the fluorescence intensity of each sample (excitation 480 nm, emission 520 nm) was measured using a BioTek Cytation 5 plate reader (Agilent). Encapsulation efficiency (EE%) was calculated according to the equation:

$$\mathrm{EE}\left(\%\right)=\frac{\left(I_{\text{Triton}}-I_{\text{Water}}\right)}{I_{\text{Triton}}}*100$$

$I_{\text{Triton}}$ = fluorescence intensity of samples mixed with 1% Triton X‐100.

$I_{\text{Water}}$ = Fluorescence intensity of samples mixed with ultra‐pure water.

### 2.9 Dynamic Light Scattering and Electrophoretic Light Scattering

DLS and electrophoretic light scattering (ELS) experiments were carried out using a Zetasizer Ultra Red (Malvern Panalytical) to determine particle size using the *Z*‐average, zeta potential and polydispersity index of dsRNA‐LPC formulations. Three consecutive measurements were conducted in backscattering mode using a DTS1070 folded capillary zeta cell (Malvern Panalytical). Measurements at elevated temperatures were preceded by a 10‐min equilibration. Results were pooled and averaged using ZS Explorer v3.3 (Malvern Panalytical) and visualized using Origin2023b.

### 2.10 Cryo‐TEM

Cryo‐transmission electron microscopy (Cryo‐TEM) samples were prepared by applying 7 μL dsRNA‐LPC to a gold grid covered with UltrAuFoil 1.2/1.3 (Quantifoil, Micro Tools, Germany). Excess liquid was blotted from the reverse of the grid using strips of filter paper. The samples were plunge‐frozen in liquid ethane (−180°C) in a Cryobox (Carl Zeiss). Excess ethane was removed with filter paper. The samples were immediately transferred to a pre‐cooled cryo‐electron microscope (Philips CM 120) using a Gatan 626 cryo‐transfer holder. The microscope was operated at 120 kV under low dose conditions. Images were recorded with an F216 2K CMOC camera (TVIPS). Four images were recorded and averaged to one image to reduce noise.

### 2.11 Spray‐on‐Plant Feeding Assays

We evaluated the bioactivity of the dsRNA‐LPC formulations against CPB using an in‐house spraying assay. Tendrils of 6‐week‐old potato plants were harvested and trimmed to carry only four trifoliates of approximately the same size. Trimmed tendrils were placed in 250‐mL flasks filled with tap water and sealed with alumina foil and Parafilm. The bottom side of the trifoliates was then sprayed three times with plain water controls, unformulated dsRNA controls, or dsRNA‐LPC formulations. The amount of treatment dispensed per spray burst was 125 μL, resulting in 375 μL per trifoliate and 1500 μL (150 μg of dsRNA) per tendril. Each treatment included 0.1% (v/v) Silwet Gold (UPL Deutschland) to ensure sufficient foliar wetting. Sprayed tendrils were left to dry and were then placed in individual net cages, ensuring that at least one trifoliate was in contact with the bottom plate. Fifteen second‐instar (L2) CPB larvae reared in‐house were carefully placed on this trifoliate. Fully prepared net cages were placed and maintained in an incubation room at 25.5°C with 62% relative humidity and a 16‐h photoperiod. Mortality in each population was monitored on Day 1 and Days 4–8. Cages were replenished with fresh, identically trimmed but untreated tendrils on Days 4 and 7. Each experiment was carried out in triplicate. Results were pooled and averaged.

## 3 Results and Discussion

### 3.1 Synthesis of Lipopeptide Surfactants

**FIGURE 1:**
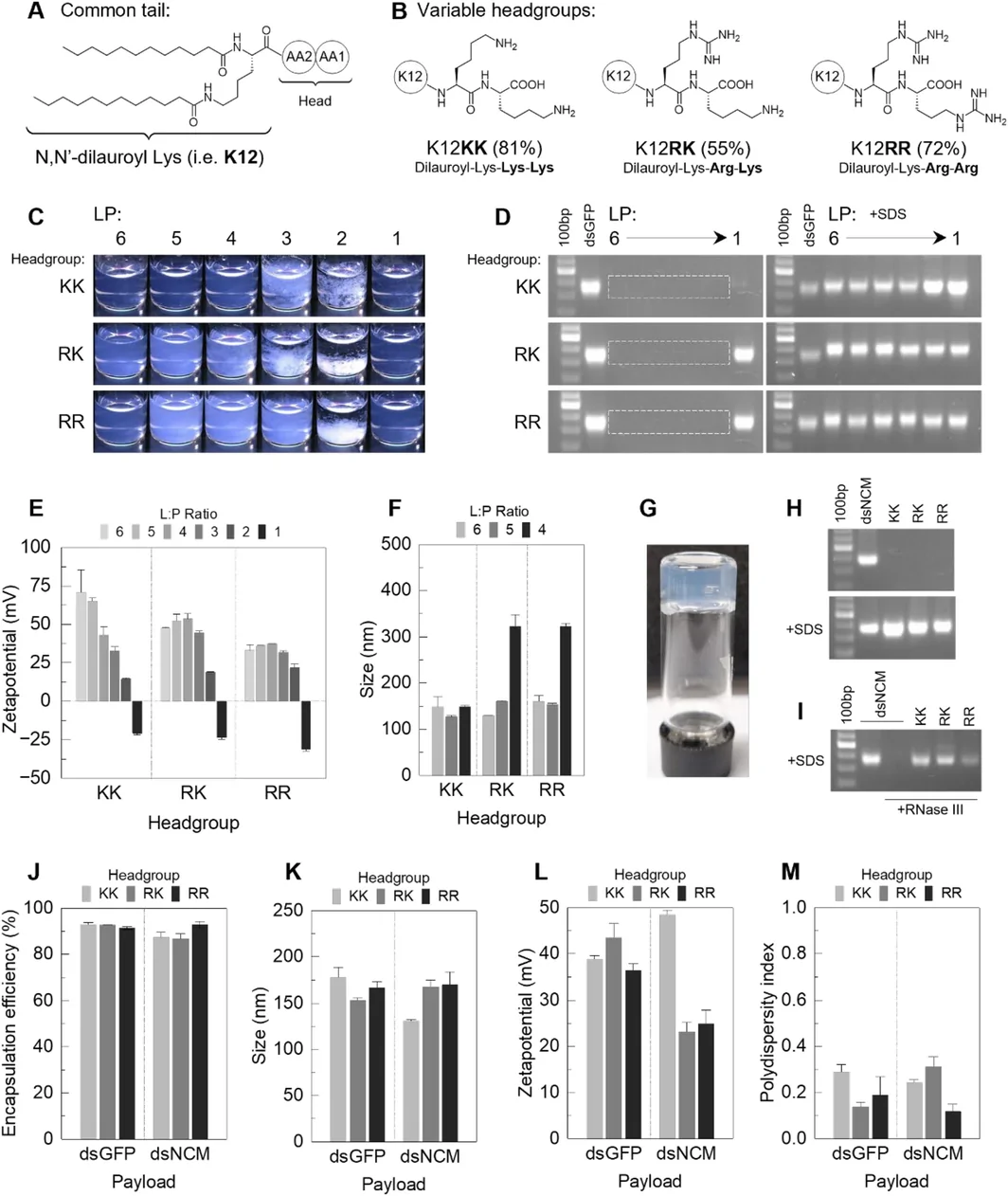
Structure–property correlations of lipopeptides and corresponding dsRNA‐lipopeptide complex (LPC) formulations. (A) Structure of the *N*,*N*′‐dilauroyl lysinyl hydrophobic tail (K12). (B) The variable head groups Lys‐Lys (KK), Arg‐Lys (RK), and Arg‐Arg (RR) were combined with the K12 tail to produce the lipopeptide surfactants K12KK, K12RK, and K12RR. (C) Visual appearance of the dsGFP‐LPC formulations prepared by rapid solvent injection at various lipid‐to‐phosphate (L:P) ratios. The formulations show different degrees of opalescence and precipitation at lower ratios. (D) Agarose gel electrophoresis of dsRNA‐LPC formulations prepared at different L:P ratios. The absence of bands corresponding to the unformulated dsGFP control indicates sufficient complexation. Addition of SDS causes decomplexation and cargo release, confirming cargo integrity. (E) Zeta potentials of dsRNA‐LPC formulations prepared for L:P ratio scouting, showing head group‐dependent trends and positive potentials down to L:P = 2. (F) Corresponding particle sizes in the L:P range of 6–4, indicating the lower stoichiometric limit of stability of dsGFP‐RK and dsGFP‐RR at L:P = 4. (G) Gelling of dsGFP‐LPC formulations (microfluidic, L:P = 5) observed after equilibration at 4°C. The gelling was reversible by equilibration at room temperature and gentle agitation. (H) Agarose gel electrophoresis of dsNCM‐LPC formulations (microfluidic, all L:P = 5) showing sufficient complexation and release following the addition of SDS. (I) The LPC formulations protect dsNCM against RNase III for up to 24 h. Unformulated dsNCM is rapidly degraded. (J) Encapsulation efficiencies of dsRNA‐LPC formulations at L:P = 5. (K–M) Particle sizes, zeta potentials and polydispersity indices of dsGFP‐LPC and dsNCM‐LPC formulations. Measurements conducted at 40°C.

Our lipopeptide surfactant design for sprayable dsRNA formulations was based on a short‐chained *N*,*N*‐dilauroyl lysinyl hydrophobic tail (Figure 1A), hereafter described as K12. The tail can be added to the N‐terminus of a growing peptide as a final condensation step during SPPS, allowing for rapid prototyping. The short‐chain dilauroyl design was chosen to ensure formulation processability. The hydrophobic double‐chain structure should facilitate lipid bilayer formation. The hydrophilic head was designed to include amino acids that do not necessarily require protonation under acidic conditions, thus providing sufficient numbers of positive charges for electrostatic complexation with the negatively charged phosphate backbone of dsRNA. The side chains of lysine (Lys, K) and arginine (Arg, R) have pKa values of 10.5 and 12.5–13.8, respectively, and are cationic under neutral pH conditions [56]. We investigated lipopeptides that feature the head groups Lys‐Lys (KK), Arg‐Lys (RK), and Arg‐Arg (RR), so the corresponding amino lipopeptides were named K12KK, K12RK, and K12RR (Figure 1B). We also synthesized a surfactant with a KR head group, but this was insoluble without additional acidification and was excluded. The head group was engineered as a dipeptide motif to compensate for intramolecular ion pair (zwitterion) formation via proton transfer and retain one cationic protonated side chain for electrostatic complexation.

The lipopeptides K12KK, K12RK, and K12RR were prepared by SPPS on a pre‐activated 2‐chlorotrityl chloride resin using the Fmoc protection scheme and HATU:DIPEA:HOBt coupling chemistry. The hydrophobic K12 tail was constructed by coupling Fmoc‐K (Fmoc)‐OH to the pre‐synthesized head groups, followed by Fmoc cleavage and endcapping with lauric acid. We carried out two rounds of endcapping to compensate for the sterically unfavorable formation of the tail. Following acidic cleavage from the resin and global deprotection of the side chains with TFA:H_2_O:TIS, we found that standard precipitation in ice‐cold diethyl ether did not yield a visually identifiable solid. We therefore removed the TFA by co‐evaporation with DCM followed by lyophilization of the residual oil. Purification by semi‐preparative RP‐HPLC achieved acceptable yields for K12KK (81%) and K12RR (72%), but a lower yield for K12RK (55%). These values underline the sterically challenging formation of the double‐chained hydrophobic tail under SPPS conditions.

### 3.2 Lipid‐to‐Phosphate Ratio of dsRNA LPC Formulations

We used rapid solvent injection formulation to evaluate the ability of the lipopeptides K12KK, K12RK, and K12RR to form complexes with dsRNA and to identify an optimal lipid‐to‐phosphate (L:P) ratio for subsequent formulation studies. The L:P ratio defines the formulation stoichiometry as the molar excess of all bilayer‐forming lipids relative to the phosphate groups in the dsRNA backbone. In addition to the commonly used nitrogen‐to‐phosphate (N:P) ratio, which represents the molar excess of cationic nitrogen atoms over phosphate groups, we use the L:P ratio as a complementary and informative descriptor of formulation composition [38]. We screened formulations across the L:P range of 6–1 (1.85–0.31 mM) for their ability to form complexes with 0.1 mg/mL of a 250‐bp dsGFP construct, covering a range of total lipid concentrations reported for other lipid‐based nucleic acid formulation systems [36, 38, 57, 58, 59, 60, 61]. The lipid blends used for complex formulation contained an 80:20 M ratio of lipopeptide to the PEGylated lipid Kolliphor HS‐15, with the total lipid amount adjusted to match the desired L:P ratio for each formulation. The 80:20 ratio was established in preliminary recipe‐screening experiments, which demonstrated that Kolliphor HS‐15 is needed to ensure stable formulations and suppress aggregation by providing steric repulsion on the bilayer surface [62] (also see Table S2). Given the blend compositions, the L:P ratios of 6–1 translate to N:P ratios of 9.6–1.6, comparable to those already reported for successful dsRNA formulations [59, 60, 61].

Figure 1C shows the visual appearance of the dsGFP‐LPCs prepared using the rapid injection method, highlighting the influence of the KK, RK, and RR head groups and L:P ratios on opalescence and stability. At high L:P ratios of 6 or 5, all lipopeptides formed stable complexes with dsGFP, leading to LPC formulations with different degrees of opalescence. At L:P = 5, complexes formed from dsGFP and K12KK (dsGFP‐KK) appeared as nearly clear solutions, whereas complexes formed from dsGFP and K12RK (dsGFP‐RK) were more opalescent, and those formed from dsGFP and K12RR (dsGFP‐RR) were cloudier in appearance. At lower L:P ratios of 4 and 3, the complexes started to precipitate, indicating the lower stoichiometric limit of stability. The dsGFP‐KK and dsGFP‐RR complexes appeared stable at L:P = 4 but precipitated at L:P = 3, whereas the dsGFP‐RK complexes precipitated even at L:P = 4. At the lowest L:P ratios of 2 and 1, no stable complexes were observed. To determine whether the precipitation resulted from insufficient complexation due to incomplete charge neutralization or heterogeneous particle formation, we analyzed the dsGFP‐LPC formulations by agarose gel electrophoresis (Figure 1D). Unformulated dsGFP migrated as a band corresponding to its expected molecular weight. The absence of corresponding bands in samples of each lipopeptide complex at L:P ratios of 6–2 indicated that charge neutralization was achieved at these ratios. At L:P = 1, insufficient charge neutralization resulted in the appearance of a band in samples of dsGFP‐RK and dsGFP‐RR. Surprisingly, dsGFP‐KK did not yield a band at this ratio, indicating that K12KK might provide sufficient charge neutralization even at low concentrations. Electrophoretic immobilization was achieved at L:P = 6–2 (N:P = 9.6–3.2), which is comparable to the N:P ratios of other cationic lipopeptide systems yet considerably lower than the N:P ratios of structurally more closely related lipopeptides [63]. The addition of sodium dodecylsulfate (SDS) to an identical set of samples and separation by gel electrophoresis resulted in the reappearance of bands of an appropriate size. The anionic surfactant SDS neutralizes the cationic charge of the lipopeptides, resulting in decomplexation and release of the dsGFP cargo, confirming dsRNA integrity and providing evidence for the electrophoretic immobilization of dsGFP in the presence of the cationic lipopeptides. Differences in migration observed between samples at different L:P ratios (and compared with the unformulated control) arise from the addition of SDS and the more or less complete neutralization of the cationic surfactants.

To conclude the L:P ratio screening, the dsGFP‐LPCs were also assessed by ELS to determine their zeta potential (Figure 1E). The highest zeta potential of 70.7 ± 15 mV was obtained from dsGFP‐KK at L:P = 6. The potentials of the dsGFP‐KK complexes declined in a near‐linear manner when approaching lower L:P ratios, reaching a negative value of −20.6 ± 1.1 at L:P = 1. Although a negative zeta potential was observed for dsGFP‐KK at L:P = 1, the formulation did not migrate toward the anode during electrophoresis, suggesting incomplete complex formation. Comparing the trends observed from the zeta potentials of dsGFP‐RK and dsGFP‐RR complexes revealed a shift from a near‐linear decrease toward lower L:P ratios to a more parabolic profile, consistent with other reported zeta potentials relative to the amounts of each lipopeptide [63]. The dsGFP‐RK and dsGFP‐RR complexes showed a slight increase of zeta potential in the order of L:P = 6 to 4, peaking at the latter with 53.5 ± 3.3 and 37.4 ± 0.2, respectively, followed by a gradual decline to −23.4 ± 1.2 and −30.9 ± 1.5 at L:P = 1. Comparing the complexes prepared from lipopeptides with different head groups showed a decrease of the average zeta potential at the higher ratios of L:P = 6 and 5 in the order of dsGFP‐KK to dsGFP‐RK to dsGFP‐RR. This trend reversed at L:P = 2 but was reestablished at L:P = 1, where dsGFP‐RR showed the most negative potential. In the mid‐range of L:P = 4 or 3, no clear trend was observed. The particle sizes measured in parallel reflect the lower stoichiometric limit of stability of dsGFP‐RK and dsGFP‐RR at L:P = 4, whereas dsGFP‐KK complexes remained stable at these ratios (Figure 1F). However, all LPCs showed positive zeta potentials in the L:P range 6–2, indicating that the precipitation observed at L:P = 4 or 3 most likely occurred due to a lack of bilayer‐forming material.

### 3.3 Microfluidic Preparation of dsRNA‐LPCs

Based on the L:P screening results, visual inspection indicated a lower stoichiometric stability threshold between L:P = 4 and 3. In combination with the high zeta potential observed for dsGFP‐KK at L:P = 6, an L:P ratio of 5 was selected and applied across all samples in subsequent microfluidic formulation experiments, equaling a total lipid concentration of 1.54 mM. To ensure controlled and reproducible preparation, dsRNA‐LPCs were generated by microfluidic mixing using a previously optimized protocol with a 3D serpentine mixer chip featuring a hydrodynamic flow‐focusing inlet geometry, enabling the reliable evaluation of physicochemical properties and bioactivity [36, 38, 64]. The aqueous phase contained either the previously used dsGFP or an insecticidal 250‐bp dsNCM construct. The latter targets the core splicing factor *ncm* in CPB and was shown to induce mortality [65, 66]. Lipopeptide complexes obtained by microfluidic mixing of these dsRNA constructs with lipid blends containing the lipopeptides K12KK, K12RK or K12RR and Kolliphor HS‐15 (80:20, L:P = 5) were equilibrated at 4°C overnight, resulting in the formation of a slightly opalescent gel (Figure 1G). However, the gels liquefied after equilibration at room temperature and gentle agitation. Agarose gel electrophoresis showed that sufficient complexation of dsGFP and dsNCM was achieved, confirming the transferability of the scouted blends to microfluidic mixing (Figure 1H). Decomplexation and release of the nucleic acid cargos in the presence of SDS confirmed the cargo integrity, which withstood the friction forces of microfluidic mixing.

The protection provided by the lipopeptide complexes against nucleolytic digestion was tested by mixing diluted aliquots of unformulated dsRNA and dsRNA‐LPCs with RNase III followed by incubation for 24 h, after which the nuclease was quenched by adding proteinase K. Analysis of the quenched reaction mixtures by agarose gel electrophoresis revealed the complete digestion of unformulated dsNCM (Figure 1I) and dsGFP (Figure S6), as indicated by the absence of a band matching the untreated control samples. Regardless of the incorporated lipopeptide, all dsRNA‐LPCs successfully protected their cargos against nuclease activity as evidenced by bands recovered by decomplexation with SDS, confirming levels of protection similar to other dsRNA formulation systems [67].

The initial head group design was intended to enable the use of the resulting lipopeptides without requiring additional protonation by acidification of the bulk medium. To quantify the success of this design, we determined the encapsulation efficiencies (EE%) achieved by microfluidic preparation of the dsGFP‐LPCs and dsNCM‐LPCs. Aliquots of each complex were diluted with either ultra‐pure water or 1% (v/v) Triton X‐100 to give intact or partially decomplexed samples, respectively. An excess of SYBR Gold was added to each sample, and after a short incubation we recorded the fluorescence of each sample at 520 nm. Calculation of the EE% based on the measured fluorescence revealed that acceptable encapsulation efficiencies were achieved with each lipopeptide blend (Figure 1J). Complexes containing dsGFP were formed with an average EE% of 92%, slightly higher than the 89% average EE% of dsNCM‐containing complexes. The high EE% not only confirms the applicability of the lysine and arginine head group design but also underlines the influence of the nucleic acid cargo during complex formation.

Samples were equilibrated at 40°C before measuring the particle sizes, zeta potentials and polydispersity indices (PDIs) at this elevated temperature, where all samples formed fully liquid dispersions. Particle size measurement revealed Z‐average values ranging from 178 ± 11 nm for dsGFP‐KK to 132 ± 1.3 nm for dsNCM‐KK, again demonstrating the influence of the cargo (Figure 1K). However, the zeta potentials differed from the values anticipated from the previous L:P screening (Figure 1L). Notably, dsGFP‐KK (65 ± 2 to 39 ± 1 mV) and dsGFP‐RK (52 ± 5 to 44 ± 3 mV) had weaker charges than predicted, whereas dsGFP‐RR (36 ± 4 to 36 ± 1 mV) remained almost identical. The zeta potential measured for dsNCM‐KK (48.5 ± 0.9) was the highest of all samples, whereas dsNCM‐RK (23.2 ± 2.0) and dsNCM‐RR (24.9 ± 3.0) showed comparably low potentials. PDIs indicate the heterogeneity of particle populations, and our DLS data revealed acceptable PDIs in the range 0.31–0.12 (Figure 1M).

### 3.4 Cryo‐Transmission Electron Microscopy

**FIGURE 2:**
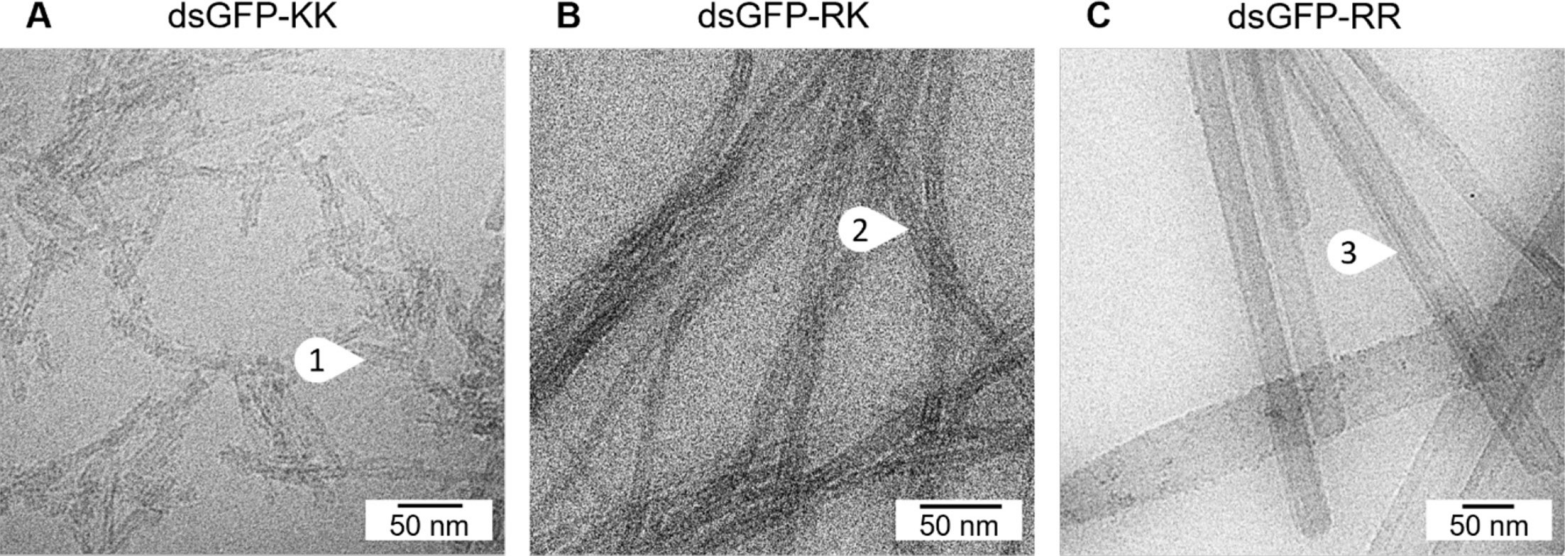
Cryo‐TEM images of dsGFP‐LPCs quenched in liquid ethane, revealing fibrillar gel‐like network structures. (A) dsGFP‐KK complexes form fibrils ~50 nm in length and 4–5 nm in width, which appear to be bent, showing a lamellar double‐fibril structure (1). (B) dsGFP‐RK complexes form networks of long and densely aggregated fibrils 4–5 nm in width. The double‐fibrillar arrangements show a lamellar structure (2). The multi‐layered ribbon‐like structures observed from the dsGFP‐RR complexes are 20–200 nm in width, and the inner lamellar structure reflects the aggregation of individual fibrils 4–5 nm in width (3).

Given the observed temperature‐dependent gelation of the dsRNA‐LPCs, a property also reported for other surfactant systems based on *N*,*N*′‐diacyl lysine [68, 69], we used cryo‐TEM to determine the morphology of the LPCs. The analysis of dsGFP‐LPC formulations (microfluidic, L:P = 5) revealed fibrillar structures, similar to the structures described for other lipopeptides [70, 71, 72]. The different head groups of the lipopeptides influenced the structure of the fibrils. The double‐lysine head group in dsGFP‐KK complexes led to short fibrils ~50 nm in length and 4–5 nm in width (Figure 2A). The replacement of one lysine with arginine in dsGFP‐RK complexes altered the fibril structure, producing a dense network of homogeneous, double‐layered fibrils 4–5 nm in width (Figure 2B). The double‐arginine head group resulted in long, ribbon‐like structures varying in width from 20 to 200 nm, probably due to multi‐layering (Figure 2C). Based on the inner lamellar structure observed for every formulation and the high encapsulation efficiencies in the SYBR Gold assay, these structures are likely to be aggregates of dsRNAs enclosed in lipopeptide bilayers. The fibrillar structures reflect the flexibility of the *N*,*N*′‐dilauroyl lysine tail, which allows parallel molecular packing of the fatty acid residues, resulting in the arrangement of surfactant units as a zero curvature bilayer along the phosphate backbone [73, 74]. At lower temperatures, these lipopeptide‐dsRNA fibrils aggregate into dense networks that mediate gelation. Higher temperatures facilitate random coiling of the fibrils. The reported particle sizes and zeta potentials are probably associated with these coils, which may consist of low numbers of fibrils. The spherical particle aggregates described for other lipopeptide‐based formulations were not observed in our samples under the conditions used for cryo‐TEM sample preparation [75, 76].

### 3.5 Sprayability and CPB Bioactivity Assay

**FIGURE 3:**
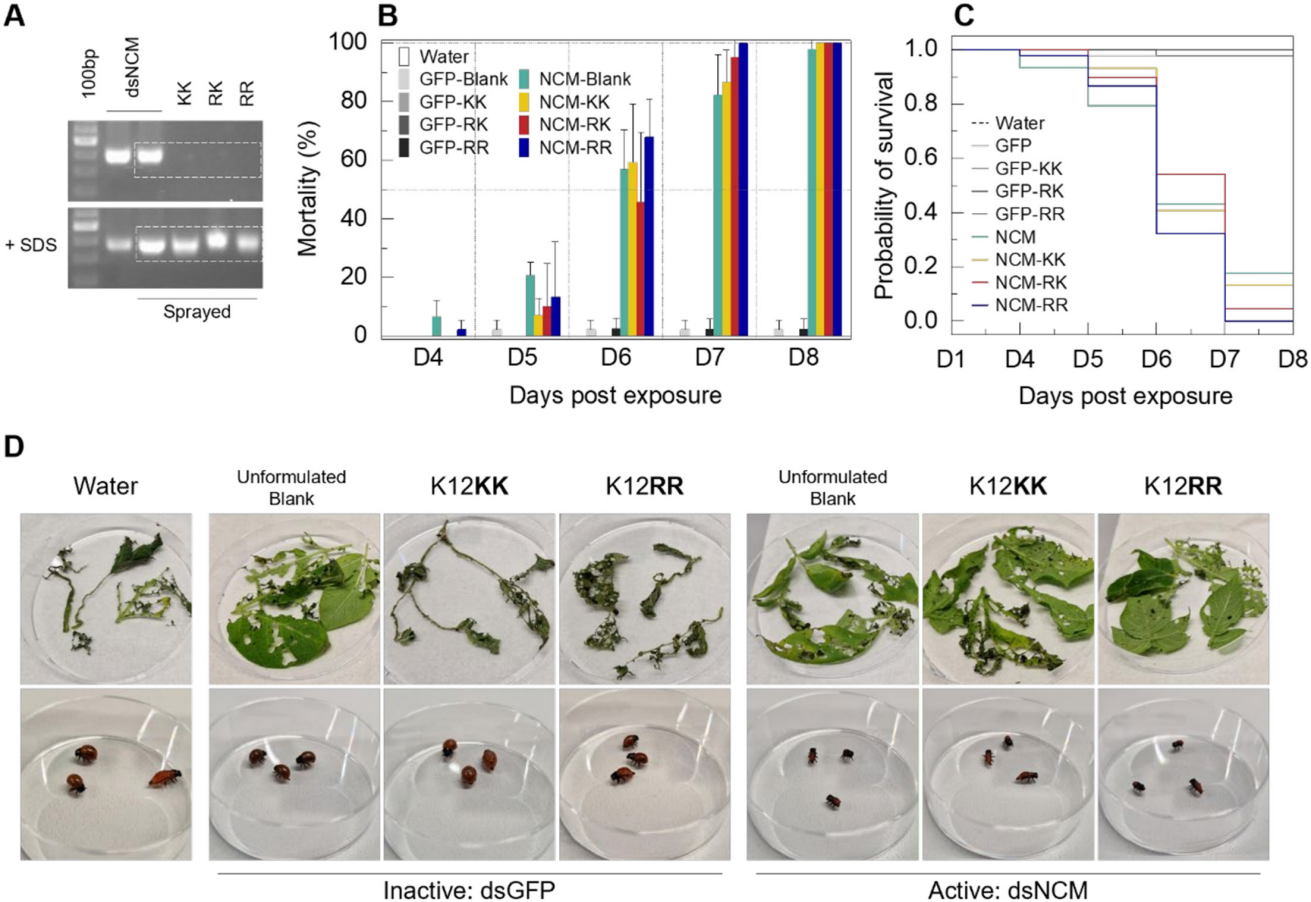
Sprayable dsRNA‐LPC formulations and CPB bioactivity assay. (A) Agarose gel electrophoresis of sprayed dsNCM‐LPC samples shows that the formulations remain stable in the presence of the wetting agent. The presence of dsNCM bands following decomplexation induced by SDS confirms the sprayability of the LPC constructs. (B) Relative mortality in the CPB assay reveals low background mortality of unformulated dsRNA and high active treatment mortality. Errors bars denote standard errors of the mean. (C) Kaplan–Meier plot shows the probability of survival, indicating the effects of short delay and more rapid progression mediated by the LPC formulations such as dsNCM‐RR. However, log‐ranked Mantel–Cox testing (*p* < 0.05) showed no statistically significant effect mediated by the LPC formulations, in comparison to unformulated dsNCM. (D) Plant material and CPB larvae collected during replenishment on Day 4. Larvae treated with either plain water or dsGFP‐containing sprays show normal growth and extensive leaf damage caused by feeding. Exposure to dsNCM‐containing sprays causes larval growth impairment and reduces the extent of leaf feeding damage (Petri dishes 100 mm in diameter are shown in all images).

RNAi‐based pest control for leaf‐feeding insects, such as CPB, requires dsRNA formulations that are both sprayable and compatible with common adjuvants. To evaluate these properties prior to bioactivity testing, unformulated dsRNA and dsRNA–LPCs were mixed with 0.1% (v/v) Silwet Gold, and samples were collected following application using handheld spray bottles. Agarose gel electrophoresis of the sprayed samples confirmed the stability of the dsRNA‐LPCs in the presence of the commercial wetting agent and integrity after decomplexation with SDS (Figure 3A).

The bioactivity of the dsGFP‐LPCs and dsNCM‐LPCs against CPB was assessed using an in‐house spraying assay. Briefly, trimmed potato plant tendrils were sprayed with treatments containing either water, unformulated dsRNA or dsRNA‐LPCs, with a total of 1500 μL (at 0.1 μg/μL) applied across four trifoliates (~150 μg dsRNA per treatment and ~37.5 μg per trifoliate). The wetting agent ensured sufficient foliar coverage. Fifteen L2 larvae of CPB were placed on one trifoliate to start the assay. The bioactivity assay was a randomized study in triplicate, monitoring the mortality of each population on Day 1 and Days 4–8. The average mortality was negligible in the water control groups (Figure 3B). Similarly, populations treated with unformulated dsGFP and dsGFP‐RR showed minimal mortality. No mortality was observed following treatment with dsGFP‐KK or dsGFP‐RK, demonstrating that the lipopeptide surfactants did not exert any significant toxic effects at the tested concentration and exposure duration. In contrast, intense mortality was observed in populations treated with unformulated dsNCM or dsNCM‐LPCs. Unformulated dsNCM induced ~50% mortality by Day 6, followed by a steady increase to near‐complete population control by Day 8. The complexes dsNCM‐KK and dsNCM‐RR led to comparable mortality rates of 50% or higher on Day 6, but dsNCM‐RK showed a slight lag. The induced mortality confirmed dsRNA cargo release after oral ingestion and is consistent with earlier reports regarding dsNCM, comparable with other CPB‐targeting dsRNA agents [31, 32, 33, 65, 66]. The mortality‐inducing effect was strongest in populations treated with dsNCM‐RR, where maximum lethal effects were observed by day 8. The Kaplan–Meier plot (Figure 3C), illustrating the survival probability of each population, supports these observations. Although an earlier onset of mortality was observed for unformulated dsNCM, populations treated with dsNCM‐RR showed a markedly reduced survival probability by day 6, exceeding the effects of unformulated dsNCM. Additionally, at the end of the observation period, populations treated with dsNCM‐RR showed a notably lower probability of survival compared to populations treated with unformulated dsNCM, demonstrating the potential of LPC formulations to modulate the bioactivity of insecticidal dsRNA agents. The enhanced bioactivity of K12RR is mechanistically not yet understood and should be interpreted cautiously. We propose that head‐group composition affects particle surface charge and, consequently, interactions with biological barriers, cellular uptake, and intracellular cargo release. K12RR may therefore provide a favorable balance of these properties rather than benefiting from a single structural feature. Figure 3D indicates the effect of dsNCM‐containing treatments on CPB growth and feeding. Insects exposed to inactive treatments (plain water or dsGFP‐containing sprays) showed unimpaired growth, confirming the absence of toxicity, and extensive leaf damage was observed. In contrast, larvae exposed to dsNCM‐containing sprays show impaired growth, indicating the successful delivery and release of the active agent, and the extent of leaf damage was much lower.

## 4 Conclusions

We have demonstrated the potential of dsRNA‐LPCs for the development of agricultural formulations that protect their dsRNA cargo and enable sprayable delivery to pest insects. To the best of our knowledge, we have demonstrated for the first time the successful delivery of dsRNA‐LPCs to pest insects via the oral route following application by spraying. To assess the scalability and long‐term stability of such formulations, a more detailed understanding of dsRNA‐LPCs is required. Further studies can elucidate the relationship between flexible and rigid hydrophobic tails on the morphology of dsRNA‐LPCs and establish comprehensive structure‐process‐activity relationships for dsRNA‐LPCs based on short‐chain lipopeptides.

## Author Contributions

P.G. designed and evaluated the experiments and prepared the figures. P.G. and C.H. drafted the manuscript. F.S. carried out the cryo‐TEM experiments and helped to draft the manuscript. A.V. and C.H. coordinated this project and helped to draft the manuscript. All authors gave final approval for publication.

## Conflicts of Interest

The authors declare no conflicts of interest.

## Supporting information


**Table S1:** Protocol used for SPPS of the amino acid surfactants.
**Figure S1:** Ultra‐high performance liquid chromatography high‐resolution tandem mass spectrometry analysis of N,N′‐dilauroyl lysinyl lysinyl lysine (K12KK). (A) Overlay of base peak chromatogram (black) and extracted ion chromatograms of [M + H]+: C42H83N6O6 (orange) and [M + 2H]2+: C42H84N6O6 (blue). (B) Identical overlay with selected retention time frame (10–15 min). (C) Overview mass spectrum obtained from integration of main peak base peak, showing [M + H]+ and (D) [M + 2H]2+ of K12KK.
**Figure S2:** Ultra‐high performance liquid chromatography high‐resolution tandem mass spectrometry analysis of N,N′‐dilauroyl lysinyl arginyl lysine (K12RK). (A) Overlay of base peak chromatogram (black) and extracted ion chromatograms of [M + H]+: C42H83N8O6 (orange) and [M + 2H]2+: C42H84N8O6 (blue). (B) Identical overlay with selected retention time frame (10–15 min). (C) Overview mass spectrum obtained from integration of main peak base peak, showing [M + H]+ and (D) [M + 2H]2+ of K12RK.
**Figure S3:** Ultra‐high performance liquid chromatography high‐resolution tandem mass spectrometry analysis of N,N′‐dilauroyl lysinyl arginyl arginine (K12RR). (A) Overlay of base peak chromatogram (black) and extracted ion chromatograms of [M + H]+: C42H83N10O6 (orange) and [M + 2H]2+: C42H84N10O6 (blue). (B) Identical overlay with selected retention time frame (10–15 min). (C) Overview mass spectrum obtained from integration of main peak base peak, showing [M + H]+ and (D) [M + 2H]2+ of K12RR.
**Table S2:** Formulation stoichiometry and DLS data of the L:P ratio screening experiments.
**Figure S4:** Overlay of particle size distributions (by intensity, number and volume) of dsGFP‐LPCs prepared by rapid injection. The overlays highlight the influence of the lipopeptide surfactant head group on the particle size population.
**Figure S5:** Correlograms of particle size distributions shown in Figure S4.
**Table S3:** Microfluidic mixing protocol.
**Table S4:** Dynamic light scattering and encapsulation efficiency assay of dsGFP‐LPCs and dsNCM‐LPCs prepared by microfluidic mixing. Measurements were conducted at 40°C.
**Figure S6:** Agarose gel electrophoresis of dsGFP‐LPC formulations (all L:p = 5) treated with RNase III for 24 h. Unformulated dsGFP is rapidly degraded, while LPCs successfully protect against enzymatic digestion, as evidenced by the recovery of appropriate bands after decomplexation using SDS.
**Figure S7:** Overlay of particle size distributions (by intensity, number and volume) of dsGFP‐LPCs and dsNCM‐LPCs prepared by microfluidic mixing. The more homogeneous appearance of the size distributions illustrates the improved quality of formulations prepared by microfluidic mixing.
**Figure S8:** Correlograms of particle size distributions shown in Figure S7.

## Data Availability

The data that support the findings of this study are available from the corresponding author upon reasonable request.

## References

[1] N. K. Arora , “Agricultural Sustainability and Food Security,” Environmental Sustainability 1, no. 3 (2018): 217–219, 10.1007/s42398-018-00032-2.

[2] J. Gaffney , J. Bing , P. F. Byrne , et al., “Science‐Based Intensive Agriculture: Sustainability, Food Security, and the Role of Technology,” Global Food Security 23 (2019): 236–244, 10.1016/j.gfs.2019.08.003.

[3] K. Pawlak and M. Kołodziejczak , “The Role of Agriculture in Ensuring Food Security in Developing Countries: Considerations in the Context of the Problem of Sustainable Food Production,” Sustainability 13 (2020): 5488, 10.3390/su12135488.

[4] D. Renault , E. Angulo , R. N. Cuthbert , et al., “The Magnitude, Diversity, and Distribution of the Economic Costs of Invasive Terrestrial Invertebrates Worldwide,” Science of the Total Environment 835 (2022): 155391, 10.1016/j.scitotenv.2022.155391. 35461930

[5] C. J. A. Bradshaw , B. Leroy , C. Bellard , et al., “Massive Yet Grossly Underestimated Global Costs of Invasive Insects,” Nature Communications 7, no. 1 (2016): 12986, 10.1038/ncomms12986. PMC5059451 27698460

[6] C. M. Oliveira , A. M. Auad , S. M. Mendes , and M. R. Frizzas , “Crop Losses and the Economic Impact of Insect Pests on Brazilian Agriculture,” Crop Protection 56 (2014): 50–54, 10.1016/j.cropro.2013.10.022.

[7] C. A. Deutsch , J. J. Tewksbury , M. Tigchelaar , et al., “Increase in Crop Losses to Insect Pests in a Warming Climate,” Science 361, no. 6405 (2018): 916–919, 10.1126/science.aat3466. 30166490

[8] P. Lehmann , T. Ammunét , M. Barton , et al., “Complex Responses of Global Insect Pests to Climate Warming,” Frontiers in Ecology and the Environment 3 (2020): 141–150, 10.1002/fee.2160.

[9] S. Skendžić , M. Zovko , I. P. Živković , V. Lešić , and D. Lešić , “The Impact of Climate Change on Agricultural Insect Pests,” Insects 5 (2021): 440, 10.3390/insects12050440. PMC8150874 34066138

[10] M. Calvo‐Agudo , J. González‐Cabrera , D. Sadutto , et al., “IPM‐Recommended Insecticides Harm Beneficial Insects Through Contaminated Honeydew,” Environmental Pollution 267 (2020): 115581, 10.1016/j.envpol.2020.115581. 33254691

[11] S. D. Eigenbrode and S. Adhikari , “Climate Change and Managing Insect Pests and Beneficials in Agricultural Systems,” Agronomy Journal 115, no. 5 (2023): 2194–2215, 10.1002/agj2.21399.

[12] M. E. S. Fernandes , F. M. Alves , R. C. Pereira , L. A. Aquino , F. L. Fernandes , and J. C. Zanuncio , “Lethal and Sublethal Effects of Seven Insecticides on Three Beneficial Insects in Laboratory Assays and Field Trials,” Chemosphere 156 (2016): 45–55, 10.1016/j.chemosphere.2016.04.115. 27160634

[13] M. A. Beketov , B. J. Kefford , R. B. Schäfer , and M. Liess , “Pesticides Reduce Regional Biodiversity of Stream Invertebrates,” Proceedings of the National Academy of Sciences 110, no. 27 (2013): 11039–11043, 10.1073/pnas.1305618110. PMC3704006 23776226

[14] L. Mamy , S. Pesce , W. Sanchez , et al., “Impacts of Neonicotinoids on Biodiversity: A Critical Review,” Environmental Science and Pollution Research 32, no. 6 (2025): 2794–2829, 10.1007/s11356-023-31032-3. 38036909

[15] M. Calvo‐Agudo , J. F. Tooker , M. Dicke , and A. Tena , “Insecticide‐Contaminated Honeydew: Risks for Beneficial Insects,” Biological Reviews 97, no. 2 (2022): 664–678, 10.1111/brv.12817. PMC9299500 34802185

[16] A. K. Chopra , M. K. Sharma , and S. Chamoli , “Bioaccumulation of Organochlorine Pesticides in Aquatic System‐An Overview,” Environmental Monitoring and Assessment 1 (2011): 905–916, 10.1007/s10661-010-1433-4. 20306340

[17] L. Tison , L. Beaumelle , K. Monceau , and D. Thiéry , “Transfer and Bioaccumulation of Pesticides in Terrestrial Arthropods and Food Webs: State of Knowledge and Perspectives for Research,” Chemosphere 357 (2024): 142036, 10.1016/j.chemosphere.2024.142036. 38615963

[18] Z. Li , “Spatiotemporal Pattern Models for Bioaccumulation of Pesticides in Common Herbaceous and Woody Plants,” Journal of Environmental Management 276 (2020): 111334, 10.1016/j.jenvman.2020.111334. 32980611

[19] S. Ray and S. T. Shaju , “Bioaccumulation of Pesticides in Fish Resulting Toxicities in Humans Through Food Chain and Forensic Aspects,” Environmental Analysis Health and Toxicology 38, no. 3 (2023): e2023017, 10.5620/eaht.2023017. PMC10613562 37853698

[20] C. Bass , I. Denholm , M. S. Williamson , and R. Nauen , “The Global Status of Insect Resistance to Neonicotinoid Insecticides,” Pesticide Biochemistry and Physiology 121 (2015): 78–87, 10.1016/j.pestbp.2015.04.004. 26047114

[21] R. Nauen and I. Denholm , “Resistance of Insect Pests to Neonicotinoid Insecticides: Current Status and Future Prospects,” Archives of Insect Biochemistry and Physiology 4 (2005): 200–215, 10.1002/arch.20043. 15756698

[22] D. Mota‐Sanchez and J. C. Wise , The Arthropod Pesticide Resistance Database (Michigan State University. Search tags: Genus [leptinotarsa], Type resistance [Field‐evolved resistances] (accessed on 18.06.2026). Available at, 2026), https://www.pesticideresistance.org.

[23] A. Fire and S. Xu , “Potent and Specific Genetic Interference by Double‐Stranded RNA in *Caenorhabditis elegans* ,” Nature 6669 (1998): 806–811, 10.1038/35888. 9486653

[24] M. J. Zotti and G. Smagghe , “RNAi Technology for Insect Management and Protection of Beneficial Insects From Diseases: Lessons, Challenges and Risk Assessments,” Neotropical Entomology 3 (2015): 197–213, 10.1007/s13744-015-0291-8. 26013264

[25] H. Zhang , H.‐C. Li , and X.‐X. Miao , “Feasibility, Limitation and Possible Solutions of RNAi‐Based Technology for Insect Pest Control,” Insect Science 20, no. 1 (2013): 15–30, 10.1111/j.1744-7917.2012.01513.x. 23955822

[26] M. R. Joga , M. J. Zotti , G. Smagghe , and O. Christiaens , “RNAi Efficiency, Systemic Properties, and Novel Delivery Methods for Pest Insect Control: What We Know So Far,” Frontiers in Physiology 7 (2016): 553, 10.3389/fphys.2016.00553. PMC5112363 27909411

[27] R. Katoch , A. Sethi , N. Thakur , and L. L. Murdock , “RNAi for Insect Control: Current Perspective and Future Challenges,” Applied Biochemistry and Biotechnology 171, no. 4 (2013): 847–873, 10.1007/s12010-013-0399-4. 23904259

[28] O. Christiaens , J. Niu , and C. N. T. Taning , “RNAi in Insects: A Revolution in Fundamental Research and Pest Control Applications,” Insects 7 (2020): 415, 10.3390/insects11070415. PMC7411770 32635402

[29] P. Bachman , J. Fischer , Z. Song , E. Urbanczyk‐Wochniak , and G. Watson , “Environmental Fate and Dissipation of Applied dsRNA in Soil, Aquatic Systems, and Plants,” Frontiers in Plant Science 11 (2020): 21, 10.3389/fpls.2020.00021. PMC7016216 32117368

[30] M. Zarrabian and S. M. Sherif , “Stability of Naked and Encapsulated dsRNA in Water and on Plant Surfaces: Key Data to Inform Environmental Safety and Toxicity Assessments,” Ecotoxicology and Environmental Safety 305 (2025): 119272, 10.1016/j.ecoenv.2025.119272. 41138451

[31] T. B. Rodrigues , S. K. Mishra , K. Sridharan , et al., “First Sprayable Double‐Stranded RNA‐Based Biopesticide Product Targets Proteasome Subunit Beta Type‐5 in Colorado Potato Beetle (*Leptinotarsa decemlineata*),” Frontiers in Plant Science 12 (2021): 728652, 10.3389/fpls.2021.728652. PMC8650841 34887882

[32] T. Rodrigues , K. Sridharan , B. Manley , D. Cunningham , and K. Narva , “Development of dsRNA as a Sustainable Bioinsecticide: From Laboratory to Field,” in Crop Protection Products for Sustainable Agriculture (American Chemical Society, 2021), 65–82, 10.1021/bk-2021-1390.ch005.

[33] L. Graser , E. R. L. Gordon , M. Jamison , et al., “Targeting the Proteasome Subunit PSMB5 by RNA Interference Induces Proteasome Dysfunction and Mortality in the Colorado Potato Beetle (*Leptinotarsa decemlineata*),” Scientific Reports 15, no. 1 (2025): 41183, 10.1038/s41598-025-28793-x. PMC12639069 41272274

[34] A. M. Vélez , K. Narva , M. Darlington , et al., “Chapter One ‐ Insecticidal Proteins and RNAi in the Control of Insects,” in Advances in Insect Physiology 65 (Elsevier, 2023), 1, 10.1016/bs.aiip.2023.09.007.

[35] R. K. Dhandapani , D. Gurusamy , and S. R. Palli , “Protamine–Lipid–dsRNA Nanoparticles Improve RNAi Efficiency in the Fall Armyworm, *Spodoptera frugiperda* ,” Journal of Agricultural and Food Chemistry 22 (2022): 6634–6643, 10.1021/acs.jafc.2c00901. 35612305

[36] M. Kaarow , L. Graser , E. Knorr , et al., “Enhancing the Delivery and Stability of Lipid Nanoparticle–dsRNA Formulations in the RNAi‐Recalcitrant Fall Armyworm (*Spodoptera frugiperda*),” Frontiers in Insect Science 6 (2026): 1770055, 10.3389/finsc.2026.1770055. PMC13062331 41970227

[37] S. Yan , B. Y. Ren , and J. Shen , “Nanoparticle‐Mediated Double‐Stranded RNA Delivery System: A Promising Approach for Sustainable Pest Management,” Insect Science 28, no. 1 (2021): 21–34, 10.1111/1744-7917.12822. 32478473

[38] P. Geisler , E. Knorr , F. Steiniger , et al., “Microfluidic Process‐Property Correlations of dsRNA Lipid Nanoparticle Formulations,” Scientific Reports 16, no. 1 (2026): 9653, 10.1038/s41598-026-44095-2. PMC13009477 41865046

[39] J. D. Wolf , M. Kurpiers , R. A. Baus , et al., “Characterization of an Amino Acid Based Biodegradable Surfactant Facilitating the Incorporation of DNA Into Lipophilic Delivery Systems,” Journal of Colloid and Interface Science 566 (2020): 234–241, 10.1016/j.jcis.2020.01.088. 32006819

[40] C. Costa , I. S. Oliveira , J. P. N. Silva , et al., “Effective Cytocompatible Nanovectors Based on Serine‐Derived Gemini Surfactants and Monoolein for Small Interfering RNA Delivery,” Journal of Colloid and Interface Science 584 (2021): 34–44, 10.1016/j.jcis.2020.09.077. 33039681

[41] K. Holmberg , F. Bilén , and R. Bordes , “Development of Amino Acid‐Based Surfactants: From Synthesis to Applications,” Current Opinion in Colloid & Interface Science 75 (2025): 101884, 10.1016/j.cocis.2024.101884.

[42] Y. Pan , W. Wang , C. Dai , et al., “Bacillus‐Derived Lipopeptides as Green Biosurfactants: Structures, Functions, Production, and Safety for Sustainable Applications,” Comprehensive Reviews in Food Science and Food Safety 25, no. 3 (2026): e70508, 10.1111/1541-4337.70508. 42132207

[43] T. M. S. Lima , L. C. Procópio , F. D. Brandão , A. M. X. Carvalho , M. R. Tótola , and A. C. Borges , “Biodegradability of Bacterial Surfactants,” Biodegradation 22, no. 3 (2011): 585–592, 10.1007/s10532-010-9431-3. 21053055

[44] I. Mnif and D. Ghribi , “Review Lipopeptides Biosurfactants: Mean Classes and New Insights for Industrial, Biomedical, and Environmental Applications,” Peptide Science 104, no. 3 (2015): 129–147, 10.1002/bip.22630. 25808118

[45] V. S. V. Santos , E. Silveira , and B. B. Pereira , “Toxicity and Applications of Surfactin for Health and Environmental Biotechnology,” Journal of Toxicology and Environmental Health 21, no. 6–8 (2018): 382–399, 10.1080/10937404.2018.1564712. 30614421

[46] D. Fei , G.‐. W. Zhou , Z.‐. Q. Yu , et al., “Low‐Toxic and Nonirritant Biosurfactant Surfactin and Its Performances in Detergent Formulations,” Journal of Surfactants and Detergents 1 (2020): 108–118, 10.1002/jsde.12356.

[47] P. S. Kumar and P. T. Ngueagni , “A Review on New Aspects of Lipopeptide Biosurfactant: Types, Production, Properties and Its Application in the Bioremediation Process,” Journal of Hazardous Materials 407 (2021): 124827, 10.1016/j.jhazmat.2020.124827. 33352424

[48] J. Garcia , Á. Fernández‐Blanco , M. Teixidó , M. Sánchez‐Navarro , and E. Giralt , “d‐Polyarginine Lipopeptides as Intestinal Permeation Enhancers,” ChemMedChem 13, no. 19 (2018): 2045–2052, 10.1002/cmdc.201800428. 30063113

[49] C. Dutta , K. Chakraborty , and R. Sinha Roy , “Engineered Nanostructured Facial Lipopeptide as Highly Efficient Molecular Transporter,” ACS Applied Materials & Interfaces 33 (2015): 18397–18405, 10.1021/acsami.5b04392. 26238518

[50] K. Sydow , V. P. Torchilin , and M. Dathe , “Lipopeptide‐Modified PEG‐PE‐Based Pharmaceutical Nanocarriers for Enhanced Uptake in Blood–Brain Barrier Cells and Improved Cytotoxicity Against Glioma Cells,” European Journal of Lipid Science and Technology 116, no. 9 (2014): 1174–1183, 10.1002/ejlt.201300373.

[51] A. Phungula , S. Zuffi , S. Thongsom , et al., “Lauryl‐NrTP6 Lipopeptide Self‐Assembled Nanorods for Nuclear‐Targeted Delivery of Doxorubicin,” Nanoscale 17, no. 8 (2025): 4659–4669, 10.1039/D4NR04068F. 39812415

[52] R. Münter , M. Bak , E. Christensen , et al., “Mechanisms of Selective Monocyte Targeting by Liposomes Functionalized With a Cationic, Arginine‐Rich Lipopeptide,” Acta Biomaterialia 144 (2022): 96–108, 10.1016/j.actbio.2022.03.029. 35314364

[53] N. A. Schilling , A. Berscheid , J. Schumacher , et al., “Synthetische Analoga Zeigen Die Essentiellen Strukturmotive von Lugdunin und Seinen Protonentransport,” Angewandte Chemie 27 (2018): 9333–9338, 10.1002/ange.201901589.

[54] M. A. Schubert and C. C. Müller‐Goymann , “Solvent Injection as a New Approach for Manufacturing Lipid Nanoparticles – Evaluation of the Method and Process Parameters,” European Journal of Pharmaceutics and Biopharmaceutics 1, no. 1 (2003): 125–131, 10.1016/S0939-6411(02)00130-3. 12551713

[55] R. El‐Mayta , M. S. Padilla , M. M. Billingsley , X. Han , and M. J. Mitchell , “Testing the In Vitro and In Vivo Efficiency of mRNA‐Lipid Nanoparticles Formulated by Microfluidic Mixing,” Journal of Visualized Experiments 191 (2023): e64810, 10.3791/64810. 36744791

[56] C. T. Armstrong , P. E. Mason , J. L. R. Anderson , and C. E. Dempsey , “Arginine Side Chain Interactions and the Role of Arginine as a Gating Charge Carrier in Voltage Sensitive Ion Channels,” Scientific Reports 1 (2016): 21759, 10.1038/srep21759. PMC4761985 26899474

[57] J. Xie , J. Zhang , J. Yang , et al., “Microfluidic‐Based dsRNA Delivery Nanoplatform for Efficient *Spodoptera exigua* Control,” Journal of Agricultural and Food Chemistry 22 (2024): 12508–12515, 10.1021/acs.jafc.4c03307. 38788129

[58] C. Su , S. Liu , M. Sun , et al., “Delivery of Methoprene‐Tolerant dsRNA to Improve RNAi Efficiency by Modified Liposomes for Pest Control,” ACS Applied Materials & Interfaces 15, no. 10 (2023): 13576–13588, 10.1021/acsami.2c20151. 36880527

[59] O. O. Koloskova , A. A. Nikonova , U. A. Budanova , et al., “Synthesis and Evaluation of Novel Lipopeptide as a Vehicle for Efficient Gene Delivery and Gene Silencing,” European Journal of Pharmaceutics and Biopharmaceutics 102 (2016): 159–167, 10.1016/j.ejpb.2016.03.014. 26992289

[60] X. L. Wang , S. Ramusovic , T. Nguyen , and Z. R. Lu , “Novel Polymerizable Surfactants With pH‐Sensitive Amphiphilicity and Cell Membrane Disruption for Efficient siRNA Delivery,” Bioconjugate Chemistry 18, no. 6 (2007): 2169–2177, 10.1021/bc700285q. 17939730

[61] X. Ma , R. Cong , X. Cui , et al., “Dendritic Lipopeptide‐Based Transdermal siRNA Delivery Systems for Effective Non‐Invasive Therapy in Psoriasis,” Journal of Controlled Release 381 (2025): 113581, 10.1016/j.jconrel.2025.113581. 40020928

[62] X. Cai , J. Zhai , N. Tran , X. Mulet , and C. J. Drummond , “Chapter Three ‐ Lipid Nanoparticle Steric Stabilization Roadmap,” in Advances in Biomembranes and Lipid Self‐Assembly, vol. 35 (Academic Press (Elsevier), 2022), 41–75, 10.1016/bs.abl.2022.05.003.

[63] Tarwadi , J. A. Jazayeri , R. J. Prankerd , and C. W. Pouton , “Preparation and In Vitro Evaluation of Novel Lipopeptide Transfection Agents for Efficient Gene Delivery,” Bioconjugate Chemistry 19, no. 4 (2008): 940–950, 10.1021/bc700463q. 18333604

[64] T. Hammoum , K. Konate , Y. Mousli , et al., “Formulation of Peptide‐Based Nanoparticles Using a Microfluidic Device,” Journal of Peptide Science 32, no. 7 (2026): e70107, 10.1002/psc.70107. PMC13250954 42267653

[65] E. Knorr , A. Billion , E. Fishilevich , et al., “Knockdown of Genes Involved in Transcription and Splicing Reveals Novel RNAi Targets for Pest Control,” Frontiers in Agronomy 3 (2021): 715823, 10.3389/fagro.2021.715823.

[66] E. Knorr , E. Fishilevich , L. Tenbusch , et al., “Gene Silencing in *Tribolium castaneum* as a Tool for the Targeted Identification of Candidate RNAi Targets in Crop Pests,” Scientific Reports 8, no. 1 (2018): 2061, 10.1038/s41598-018-20416-y. PMC5794766 29391456

[67] B. W. Moorlach , A. R. Sede , K. M. Hermann , et al., “Interpolyelectrolyte Complexes of In Vivo Produced dsRNA With Chitosan and Alginate for Enhanced Plant Protection Against Tobacco Mosaic Virus,” International Journal of Biological Macromolecules 306 (2025): 141579, 10.1016/j.ijbiomac.2025.141579. 40023414

[68] Q. Li , J. Zhang , G. Zhang , and B. Xu , “L‐Lysine‐Based Gelators for the Formation of Oleogels in Four Vegetable Oils,” Molecules 4 (2022): 1369, 10.3390/molecules27041369. PMC8876487 35209157

[69] M. Suzuki , T. Abe , and K. Hanabusa , “Low‐Molecular‐Weight Gelators Based on Nα‐Acetyl‐Nε‐Dodecyl‐L‐Lysine and Their Amphiphilic Gelation Properties,” Journal of Colloid and Interface Science 341, no. 1 (2010): 69–74, 10.1016/j.jcis.2009.09.010. 19846106

[70] E. Rosa , L. de Mello , V. Castelletto , et al., “Cell Adhesion Motif‐Functionalized Lipopeptides: Nanostructure and Selective Myoblast Cytocompatibility,” Biomacromolecules 24, no. 1 (2023): 213–224, 10.1021/acs.biomac.2c01068. PMC9832505 36520063

[71] L. Ziserman , H. Y. Lee , S. R. Raghavan , A. Mor , and D. Danino , “Unraveling the Mechanism of Nanotube Formation by Chiral Self‐Assembly of Amphiphiles,” Journal of the American Chemical Society 133, no. 8 (2011): 2511–2517, 10.1021/ja107069f. 21244023

[72] I. W. Hamley , A. Dehsorkhi , and V. Castelletto , “Self‐Assembled Arginine‐Coated Peptide Nanosheets in Water,” Chemical Communications 49, no. 18 (2013): 1850–1852, 10.1039/C3CC39057H. 23360959

[73] O. G. Mouritsen , “Lipids, Curvature, and Nano‐Medicine,” European Journal of Lipid Science and Technology 113, no. 10 (2011): 1174–1187, 10.1002/ejlt.201100050. PMC3229985 22164124

[74] M. F. Brown , “Curvature Forces in Membrane Lipid‐Protein Interactions,” Biochemistry 51, no. 49 (2012): 9782–9795, 10.1021/bi301332v. PMC5176250 23163284

[75] M. S. Alqahtani , R. Syed , A. S. Alqahtani , O. M. Almarfadi , M. A. Roni , and S. S. Sadhu , “Synthesis and Bioactivity of a Novel Surfactin‐Based Lipopeptide for mRNA Delivery,” Nanoscale Advances 6, no. 20 (2024): 5193–5206, 10.1039/D4NA00404C. PMC11376094 39247856

[76] H. Xiao , B. Feng , D. Gao , et al., “A Naturally Derived Lipopeptide Lipid Nanoparticle Platform Enabling Multiple Nucleic Acids Delivery,” Bioactive Materials 54 (2025): 829–849, 10.1016/j.bioactmat.2025.09.007. PMC12628137 41268339

