# Supplementary material for: Design of Lipopeptides and dsRNA‐Lipopeptide Complexes for Sprayable RNAi‐Based Plant Protection
Source: J Pept Sci. 2026 Sep 25;32(11):e70133. doi: 10.1002/psc.70133 (PMC13613245; doi:10.1002/psc.70133)
Supplement: Supplementary file 1 [file PSC-32-e70133-s001.docx]

**Design of lipopeptides and dsRNA-lipopeptide complexes for sprayable RNAi-based plant protection**

Pascal Geisler^1^, Frank Steininger^2^, Andreas Vilcinskas^1,3^ & Christoph Hellmann^1^

^1^ Fraunhofer Institute for Molecular Biology and Applied Ecology IME, 35392 Giessen, Germany

^2^ Electron Microscopy Center, Jena University Hospital, Friedrich Schiller University Jena, 07743 Jena, Germany

^3^ Institute of Insect Biotechnology, Justus-Liebig-University, 35392 Gießen, Germany

Email Corresponding author: [christoph.hellmann@ime.fraunhofer.de](mailto:christoph.hellmann@ime.fraunhofer.de)

**Double-stranded RNA**

The dsRNA used in this study was synthesized by Genolution and handled according to the manufacturer’s recommendations.

The 250-bp construct termed dsGFP targets *GFP* mRNA and was used as an inactive control in the assessment of biological activity. The sequence of dsGFP is shown below:

CCAGATCATATGAAACGGCATGACTTTTTCAAGAGTGCCATGCCCGAAGGTTATGTACAGGAAAGAACTATATTTTTCAAAGATGACGGGAACTACAAGACACGTGCTGAAGTCAAGTTTGAAGGTGATACCCTTGTTAATAGAATCGAGTTAAAAGGTATTGATTTTAAAGAAGATGGAAACATTCTTGGACACAAATTGGAATACAACTATAACTCACACAATGTATACATCATGGCAGACAAACAAA

The 250-bp construct termed dsNCM targets *ncm* mRNA, encoding the core splicing factor in CPB. The sequence of dsNCM is shown below:

TCAGGGTCTGGATCTGAAGGTTCTGAAGACGGTTCCGAAGACGATGAAGAAGATGAAGAAGTGAAGGATCAAGGAAAAATAATAGACAATACCGAAACTAATTTGATAGTTTTGAGAAGGACAATATATCTTACAATACAGTCTAGTTTGGATTTTGAAGAATGTGCTCACAAACTCCTAAAAATGGAACTCAAACCAGGACAGGAAATAGAACTCTGTCATATGTTTTTAGATTGTTGTGCCGAACAGC

**Solid-phase peptide synthesis**

The lipopeptides K12KK, K12RK and K12RR were prepared using the SPPS protocol shown in Table S1. The charged syringe reactors were agitated using a customized shaking table.

**Table S1**: Protocol used for SPPS of the amino acid surfactants.

| 1. | Charging of 10-mL syringe reactors, equipped with PE frit and blunted needles (Carl Roth),  with pre-activated 2CTC resin (0.1g/syringe). |
| --- | --- |
| 2. | Swelling of the resin (DCM, 40 mL/g, 90 min). |
| 3. | Initial functionalization according to target sequence (1.5 eq. Fmoc-amino acid, 3.0 eq. DIPEA, in dichloromethane, 20 mL/g, 90 min). |
| 4. | Washing (DCM, 3$\times$20 mL/g) and solvent exchange (DMF, 3$\times$20 mL/g) |
| 5. | N-terminal deprotection (piperidine:DMF, 20% v/v, 2$\times$40 mL/g, 5 min each). |
| 6. | Washing (DMF, 3$\times$40 mL/g). |
| 7. | Amino acid coupling according to target sequence (3 eq. Fmoc-amino acid, 3 eq. HATU, 6 eq. DIPEA, 0.1 eq. HOBt, DMF, 10 mL/g, 60 min). |
| 8. | Washing (DMF, 3$\times$20 mL/g). |
| 9. | Repetition of coupling cycle (5. – 8.) until target sequence is achieved. |
| 10. | N-terminal deprotection (piperidine:DMF, 20% v/v, 2$\times$40 mL/g, 5 min each). |
| 11. | Endcapping (3 eq. lauric acid, 3 eq. HATU, 6 eq. DIPEA, 0.1 eq. HOBt, DMF, 20 mL/g, 2$\times$60 min). |
| 12. | Washing (DMF, then isopropanol, then diethyl ether, 3$\times$20 mL/g) and drying *in vacuo* (60 min). |
| 13. | Cleavage and global deprotection (TFA: H_2_O:TIS, 95:2.5:2.5 v/v/v, 2$\times$20 mL/g, 30 min each). |
| 14. | Co-evaporation of TFA with DCM (3$\times$15–20 mL). |
| 15. | Dilution with ultra-pure water, lyophilization and chromatographic workup. |

***N*,*N*′-dilauroyl lysinyl lysinyl lysine (K12KK)**

**Sequence:**
Fmoc-Lys(Boc)-OH 🡪 Fmoc-Lys(Boc)-OH 🡪 Fmoc-Lys(Fmoc)-OH

Resin: 2-chlorotrityl chloride (1 mmol/g, 0.1 g)
Yield: 62 mg, 81 µmol, 81%

**C_42_H_82_N_6_O_6_** (766.63 g/mol)

[M+H]^+^ : C_42_H_83_N_6_O_6_, calc.: 767.6369, meas.: 767.6356, $\text{∆}$ppm = –1.69
[M+2H]^2+^ : C_42_H_84_N_6_O_6_, calc.: 384.3221, meas.: 384.3219, $\text{∆}$ppm = –0.52

**Figure S1: Ultra-high performance liquid chromatography high-resolution tandem mass spectrometry analysis of *N*,*N*′-dilauroyl lysinyl lysinyl lysine (K12KK).** (A) Overlay of base peak chromatogram (black) and extracted ion chromatograms of [M+H]^+^ : C_42_H_83_N_6_O_6_ (orange) and [M+2H]^2+^ : C_42_H_84_N_6_O_6_ (blue). (B) Identical overlay with selected retention time frame (10–15 min). (C) Overview mass spectrum obtained from integration of main peak base peak, showing [M+H]^+^ and (D) [M+2H]^2+^ of K12KK.

***N*,*N*′-dilauroyl lysinyl arginyl lysine (K12RK)**

**Sequence:**
Fmoc-Lys(Boc)-OH 🡪 Fmoc-Arg(Pbf)-OH 🡪 Fmoc-Lys(Fmoc)-OH 🡪 2$\times$Lauric acid

Resin:	2-chlorotrityl chloride (1 mmol/g, 0.1 g)
Yield:	44 mg, 55 µmol, 55%

**C_42_H_82_N_8_O_6_** (794.64 g/mol)

[M+H]^+^ : C_42_H_83_N_8_O_6_, calc.: 795.6430, meas.: 795.6427, $\text{∆}$ppm = –0.38
[M+2H]^2+^ : C_42_H_84_N_8_O_6_, calc.: 398.3251, meas.: 398.3257, $\text{∆}$ppm = 2.15

**Figure S2: Ultra-high performance liquid chromatography high-resolution tandem mass spectrometry analysis of *N*,*N*′-dilauroyl lysinyl arginyl lysine (K12RK).** (A) Overlay of base peak chromatogram (black) and extracted ion chromatograms of [M+H]^+^ : C_42_H_83_N_8_O_6_ (orange) and [M+2H]^2+^ : C_42_H_84_N_8_O_6_ (blue). (B) Identical overlay with selected retention time frame (10–15 min). (C) Overview mass spectrum obtained from integration of main peak base peak, showing [M+H]^+^ and (D) [M+2H]^2+^ of K12RK.

***N*,*N*′-dilauroyl lysinyl arginyl arginine (K12RR)**

**Sequence:**
Fmoc-Arg(Pbf)-OH 🡪 Fmoc-Arg(Pbf)-OH 🡪 Fmoc-Lys(Fmoc)-OH 🡪 2$\times$Lauric acid

Resin:	2-chlorotrityl chloride (1 mmol/g, 0.1 g)
Yield:	59 mg, 72 µmol, 72%

**C_42_H_82_N_10_O_6_** (822.64 g/mol)

[M+H]^+^ : C_42_H_83_N_10_O_6_, calc.: 823.6492, meas.: 823.6492, $\text{∆}$ppm = 0
[M+2H]^2+^ : C_42_H_84_N_10_O_6_, calc.: 412.3282, meas.: 412.3283, $\text{∆}$ppm = 0.24

**Figure S3: Ultra-high performance liquid chromatography high-resolution tandem mass spectrometry analysis of *N*,*N*′-dilauroyl lysinyl arginyl arginine (K12RR).** (A) Overlay of base peak chromatogram (black) and extracted ion chromatograms of [M+H]^+^ : C_42_H_83_N_10_O_6_ (orange) and [M+2H]^2+^ : C_42_H_84_N_10_O_6_ (blue). (B) Identical overlay with selected retention time frame (10–15 min). (C) Overview mass spectrum obtained from integration of main peak base peak, showing [M+H]^+^ and (D) [M+2H]^2+^ of K12RR.

**Table S2:** Formulation stoichiometry and DLS data of the L:P ratio screening experiments.

| **Formulation composition^1,2)^** Lipopeptide:PEG-lipid | | **Dynamic light scattering results in respect to lipopeptide** | | | | | |
| --- | --- | --- | --- | --- | --- | --- | --- |
|  |  | K12KK | | K12RK | | K12RR | |
| Total L:P (mM) | Internal L:P ratio (mM) | Z-Average (nm) | Zetapot. (mV) | Z-Average (nm) | Zetapot. (mV) | Z-Average (nm) | Zetapot. (mV) |
| 6.0 (1.85) | 4.8:1.2 (1.48:0.37) | 148 $\pm22$ | 70.7 $\pm$ 15 | 123 $\pm$ 1.2 | 47.7 $\pm$ 0.7 | 160 $\pm$ 13 | 33.1 $\pm$ 3.6 |
| 5.0 (1.54) | 4.0:1.0 (1.23:0.31) | 127 $\pm$ 2.7 | 65.1 $\pm$ 2.1 | 160 $\pm$ 1.6 | 52.2 $\pm$ 4.5 | 153 $\pm$ 3.8 | 36.1 $\pm$ 3.6 |
| 4.0 (1.23) | 3.2:0.8 (0.98:0.25) | 149 $\pm$ 2.9 | 43.2 $\pm$ 5.2 | 323 $\pm$ 25 | 53.5 $\pm$ 3.3 | 322 $\pm$ 6.5 | 37.4 $\pm$ 0.2 |
| 3.0 (0.99) | 2.4:0.6 (0.79:0.20) | n.a. | 32.9 $\pm$ 2.5 | n.a. | 44.4 $\pm$ 1.3 | n.a. | 31.8 $\pm$ 1.2 |
| 2.0 (0.62) | 1.6:0.4 (0.49:0.13) | n.a. | 14.6 $\pm$ 0.8 | n.a. | 19.1 $\pm$ 0.4 | n.a. | 21.8 $\pm$ 2.4 |
| 1.0 (0.31) | 0.8:0.2 (0.25:0.06) | n.a. | –20.6 $\pm$ 1.1 | n.a. | –23.4 $\pm$ 1.2 | n.a. | –30.9 $\pm$ 1.5 |

1) Each formulation comprises approximately 0.62 µM dsGFP, equal to 0.31 mM phosphate groups or L:P = 1.

2) The individual concentrations are calculated for the formulations after rapid injection.

**Figure S4:** Overlay of particle size distributions (by intensity, number and volume) of dsGFP-LPCs prepared by rapid injection. The overlays highlight the influence of the lipopeptide surfactant head group on the particle size population.

**Figure S5:** Correlograms of particle size distributions shown in Figure S4.

**Table S3:** Microfluidic mixing protocol.

| Step | Procedure for TFR = 12 mL/min, flow rate ratio (FRR) = Aq.:Org. = 9:1. |
| --- | --- |
| Setup cycle: | |
| 1. | Use 10-mL Luer-Slip (ID 20 mm) syringes (7644128; TH. GEYER)  to aspirate ultra-pure water and ethanol (20 mL each) into individual syringes.  Label properly as “Aq. Wash” and “Org. Wash”. |
| 2. | Connect aq. wash syringe to outer channels and org. wash syringe to central channel. Mount to pumps.  Set aq. pump to 10.8 mL/min and org. pump to 1.2 mL/min. |
| 3. | Start org. pump and run until ethanol elutes from exit tubing. Start aq. pump and run until aq. syringe is empty.  Stop both pumps and disconnect both syringes. |
| 4. | Connect empty syringes and manually purge the system with air.  Disconnect both syringes. |
| Formulation cycle: | |
| 5. | Immerse tubes containing aq. and org. phases for the experiment in a water bath and equilibrate at 65 °C for 30 min. |
| 6. | Use 10-mL Luer-Lock (ID 20 mm) syringes (7672432; TH. GEYER)  to quickly aspirate the aq. and org. phases to be mixed into individual syringes.  Purge syringes of air bubbles if necessary. A single bubble no larger than 1 mm diameter is tolerable. |
| 7. | Connect aq. phase syringe to outer channels and org. phase syringe to central channel. Mount to pumps. |
| 8. | Start org. pump and run until org. phase elutes from the exit tubing.  Start aq. pump and start collecting in desired fraction volumes as soon as the exit stream is constant. |
| 9. | Stop aq. pump and let org. pump run to roughly clean the system.  Stop both pumps and disconnect both syringes. |
| Repeat steps 4–9 for consecutive formulation experiments. | |
| Repeat steps 4 and 9 twice after last experiment. | |

**Table S4**: Dynamic light scattering and encapsulation efficiency assay of dsGFP-LPCs and dsNCM-LPCs prepared by microfluidic mixing. Measurements were conducted at 40 °C.

| Formulation | | Dynamic light scattering results | | | Encaps. Eff. |
| --- | --- | --- | --- | --- | --- |
| Blend (L:P) (mM) | Cargo^1)^ and abbreviation | Z-Average (nm) | Zetapot. (mV) | PDI | EE% |
| K12**KK**:KHS15 4.0:1.0 (1.23:0.31) | dsGFP i.e. dsGFP-KK | 178 $\pm$ 11 | 38.8 $\pm$ 0.9 | 0.29 $\pm$ 0.03 | 93 $\pm$ 1.0 |
|  | dsNCM i.e. dsNCM-KK | 132 $\pm$ 1.3 | 48.5 $\pm$ 0.9 | 0.24 $\pm$ 0.01 | 87 $\pm$ 2.2 |
| K12**RK**:KHS15 4.0:1.0 (1.23:0.31) | dsGFP i.e. dsGFP-RK | 153 $\pm$ 3.2 | 43.6 $\pm$ 3.1 | 0.14 $\pm$ 0.02 | 93 $\pm$ 0.3 |
|  | dsNCM i.e. dsNCM-RK | 168 $\pm$ 7.4 | 23.2 $\pm$ 2.0 | 0.31 $\pm$ 0.04 | 87 $\pm$ 2.0 |
| K12**RR**:KHS15 4.0:1.0 (1.23:0.31) | dsGFP i.e. dsGFP-RR | 167 $\pm$ 6.2 | 36.4 $\pm$ 1.5 | 0.19 $\pm$ 0.08 | 91 $\pm$ 0.6 |
|  | dsNCM i.e. dsNCM-RR | 170 $\pm$ 14 | 24.9 $\pm$ 3.0 | 0.12 $\pm$ 0.03 | 93 $\pm$ 1.3 |

1) Each formulation comprises approximately 0.62 µM dsRNA, equal to 0.31 mM phosphate groups or L:P = 1.

**Figure S6:** Agarose gel electrophoresis of dsGFP-LPC formulations (all L:P = 5) treated with RNase III for 24h. Unformulated dsGFP is rapidly degraded, while LPCs successfully protect against enzymatic digestion, as evidenced by the recovery of appropriate bands after decomplexation using SDS.

**Figure S7:** Overlay of particle size distributions (by intensity, number and volume) of dsGFP-LPCs and dsNCM-LPCs prepared by microfluidic mixing. The more homogeneous appearance of the size distributions illustrates the improved quality of formulations prepared by microfluidic mixing.

**Figure S8:** Correlograms of particle size distributions shown in Figure S7.
